# The interaction of interleukin-8 and PTEN inactivation promotes the malignant progression of head and neck squamous cell carcinoma via the STAT3 pathway

**DOI:** 10.1038/s41419-020-2627-5

**Published:** 2020-05-29

**Authors:** Qiaoshi Xu, Hailong Ma, Hanyue Chang, Zhien Feng, Chenping Zhang, Xi Yang

**Affiliations:** 1 Department of Oral Maxillofacial-Head and Neck Oncology, Shanghai Ninth People’s Hospital, College of Stomatology, Shanghai Jiao Tong University School of Medicine, No 639, Zhizaoju Rd, Shanghai, 200011 China; 2 National Clinical Research Center for Oral Diseases, Shanghai, 200011 China; 3 Shanghai Key Laboratory of Stomatology & Shanghai Research Institute of Stomatology, Shanghai, 200011 China; 4 Key Laboratory of Oral Medicine, Guangzhou Institute of Oral Disease, Stomatology Hospital of Guangzhou Medical University, Guangzhou, 510140 China; 5 Department of Oral and Maxillofacial-Head and Neck Oncology, Beijing Stomatological Hospital, Capital Medical University, Beijing, 100050 China

**Keywords:** Biomarkers, Genetics research

## Abstract

Interleukin-8 (IL-8) expression correlates with poor prognosis in many cancers, including head and neck squamous cell carcinoma (HNSCC), but the underlying mechanism is poorly understood. In this study, we found that overexpression of IL-8 correlated with poor outcome in HNSCC patients. IL-8 significantly increased cellular proliferation, migration, and invasion ability both in vitro and in vivo, which could be blocked by a CXCR1/2 inhibitor. IL-8 promoted the expression of MMP2, MMP9, snail, and vimentin in HNSCC cells. Furthermore, IL-8 could inactivate PTEN via phosphorylation, and then inactivated PTEN affected the phosphorylation of STAT3. Recombinant PTEN that internalized in cytoplasm decreased the expression of phosphorylated STAT3, while knockdown of PTEN led to the increased expression of phosphorylated STAT3. A STAT3 inhibitor could reverse the upregulation of invasion-associated proteins mediated by IL-8 stimulation. Furthermore, overexpression of snail and inactivated PTEN jointly promoted the autocrine effect of IL-8 on tumor cells. Last, there were positive correlations between IL-8 and snail, vimentin expression in HNSCC tissues. In summary, our study demonstrates that PTEN acts as a novel “molecular switch” to regulate IL-8/STAT3 signaling, promoting the progression of HNSCC, and indicating that this pathway may be a potential therapeutic target for HNSCC.

## Introduction

Head and neck squamous cell carcinoma (HNSCC), a common type of cancer, can cause both psychological and physiological damage to patients^1^. Tumor metastasis is the main reason for poor outcome^2^. An increasing number of cytokines have been demonstrated to be involved in tumor metastasis^3,4^. Previous studies have demonstrated that interleukin-8 (IL-8) plays an important role in the development of malignant tumors^5,6^. IL-8 was first discovered by Kownatzki in 1986 for its function of attracting human neutrophilic granulocytes^7^. CXCR1 and CXCR2 act as the receptors for IL-8 present on the cell surface; CXCR1 is the major receptor on tumor cells^8,9^. As a member of the CXC chemokine family, IL-8 is primarily secreted by tumor cells and stromal cells, such as macrophages and epithelial cells. Accumulating data have revealed that a special physicochemical environment enhances the secretion of IL-8, such as hypoxia and acidity^10^; however, studies on the effect of biomolecules in the tumor microenvironment on IL-8 secretion are lacking. Previous studies have shown the effect of IL-8 in many types of cancer, including HNSCC (refs. ^11–14^). Christofakis et al. demonstrated that IL-8 enhanced cell proliferation and migration in HNSCC (ref. ^15^). Nonetheless, the upstream source and the molecular mechanism of IL-8 in HNSCC are still obscure.

The gene of phosphate and tension homology deleted on chromosome ten (PTEN) has gained increasing amounts of attention due to its pivotal role in the tumor progression^16,17^. The deletion and inactivation of PTEN is a key factor for the occurrence and development of many cancers^18,19^. Although both IL-8 and inactivated PTEN are cancer drivers, studies on the relationship between IL-8 and PTEN are lacking. Moreover, current studies tend to believe that the main downstream pathway of PTEN is the PI3K/AKT pathway^20,21^, yet the regulatory relationship of PTEN and another critical oncogene, signal transducer and activator of transcription 3 (STAT3), is poorly explored in HNSCC.

In the present study, we found that IL-8 promoted the malignant progression of HNSCC through the STAT3 pathway, which can be blocked by a CXCR1/2 repressor. More importantly, we demonstrated that IL-8 facilitated the phosphorylation of PTEN, which could lead to the activation of STAT3. PTEN inactivation has a strong impact on the activation of STAT3 pathway. The inactivation of PTEN, increased snail expression and autocrine IL-8 can create a novel positive feedback loop that drives malignant tumor progression. These results not only indicate IL-8 as an oncogenic cytokine, but also reveal that PTEN acts as a novel “molecular switch” to regulate the IL-8/STAT3 signaling, which may be a potential therapeutic target for HNSCC.

## Results

### IL-8 is upregulated in HNSCC and correlates with poor prognosis

**Fig. 1:**
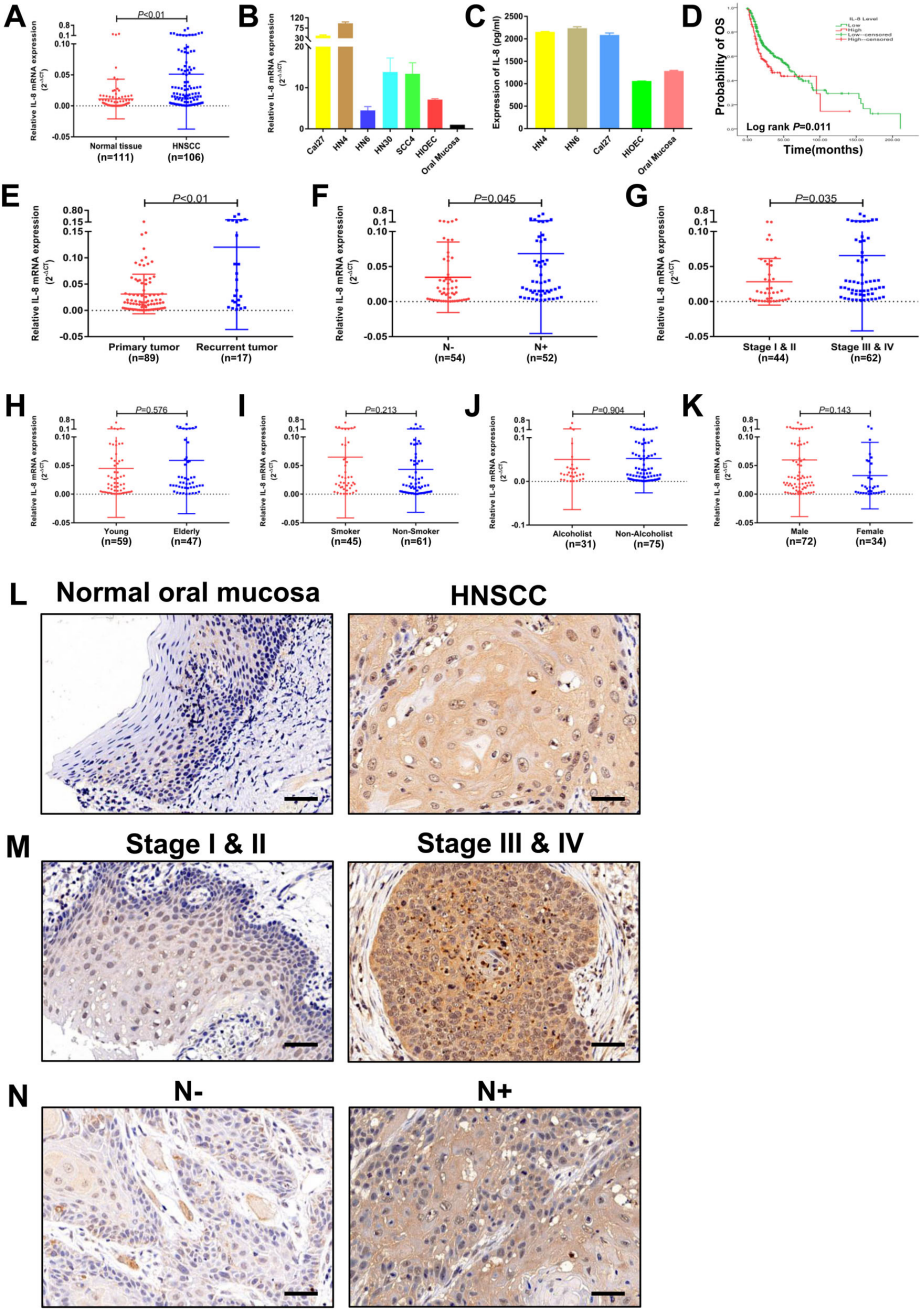
IL-8 is upregulated in HNSCC and correlates with poor prognosis. **a** Relative IL-8 mRNA expression was detected using PCR in HNSCC patients, including primary and recurrent tumors (*n* = 106), and healthy controls (*n* = 111). **b** Relative IL-8 mRNA expression was detected in HNSCC cell lines, normal oral mucosa, and HIOEC. **c** Expression of IL-8 by HN4, HN6, Cal27, HIOEC, and normal oral mucosa detected by ELISA. **d** Survival analysis was performed in the HNSCC dataset from the TCGA database. The correlation between IL-8 expression and tumor recurrence (**e**), lymph node metastasis (**f**), TNM stage (**g**), age (**h**), smoking (**i**), alcohol use (**j**), and gender (**k**) was analyzed in HNSCC patients. Representative IHC images of IL-8 correlation with tumor status (**l**), advanced stage (**m**), and lymph node metastasis (**n**) are shown (magnification 400×). Scale bar, 50 μm; *P < 0.05, **P < 0.01.

To determine the expression of IL-8 in HNSCC, 106 patients and 111 normal volunteers were enrolled in this study. Tissue samples from HNSCC and normal oral mucosa were examined by real-time PCR (RT-PCR). The results showed that HNSCC patients have significant higher expression of IL-8 than did healthy controls (Fig. 1a, *P* < 0.01). Moreover, we examined the mRNA level of IL-8 in HNSCC cell lines, as well as human immortalized oral epithelial cells (HIOECs) and primary oral keratinocytes. All HNSCC cell lines had a higher level of IL-8 than normal oral epithelial cells (Fig. 1b). Enzyme-linked immunosorbent assay (ELISA) was performed to prove this conclusion on the protein level. The secretion of IL-8 was significant higher in HNSCC cell lines compared with HIOEC and normal oral mucosa (Fig. 1c). Among those cell lines, HN6 has the lowest mRNA level of IL-8, and HN4 has the highest, followed by Cal27. However, the secretion level of IL-8 was similar between those three cell lines. To fully study the function of IL-8, we chose HN4, HN6, and Cal27 for further experiments. Survival data from The Cancer Genome Atlas (TCGA) dataset shows that HNSCC patients with a high level of IL-8 have a significantly worse outcome than patients with a low level of IL-8 (Fig. 1d, *P* = 0.011). In addition, the expression level of IL-8 also correlates with tumor recurrence (Fig. 1e, *P* < 0.01), cervical lymphatic metastasis (Fig. 1f, *P* = 0.045), and advanced stage (Fig. 1g, *P* = 0.035), but does not correlate with age, smoking, drinking, or gender (Fig. 1h–k, respectively). The above results have also been confirmed by immunohistochemical (IHC) staining in HNSCC tissue (Fig. 1l–n). These data indicate that overexpression of IL-8 correlates with aggressive disease behavior in HNSCC patients.

### IL-8 promotes malignant progression in HNSCC cell lines through CXCR1/2

**Fig. 2:**
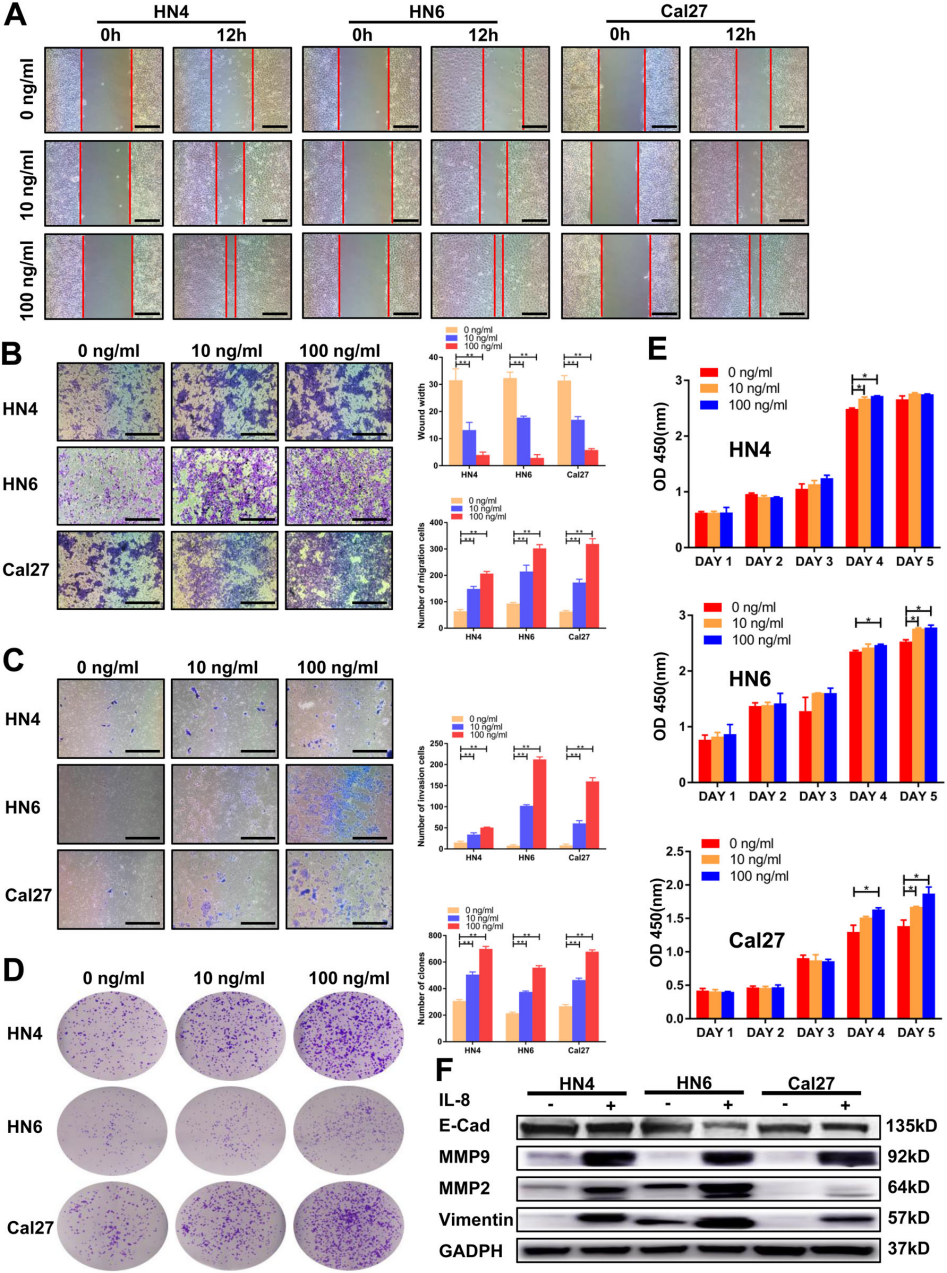
IL-8 promotes malignant progression in HNSCC cell lines. **a** Migration ability of HNSCC cells treated with the indicated IL-8 concentration was detected by a wound healing assay. Scale bar, 100 μm. **b**, **c** Transwell assays were performed to determine the migration and invasion ability of cells with different concentrations of IL-8. Scale bar, 250 μm. **d** Colony formation ability of cells in three groups was determined using a colony-forming assay. **e** The CCK8 assay was used to detect the proliferation ability of HNSCC cells with different concentrations of IL-8. **f** E-cadherin, MMP2, MMP9, and vimentin were detected using western blot after 100 ng/ml IL-8 stimulation; *P < 0.05, **P < 0.01.

To further explore the mechanism underlying poor outcome of patients with higher IL-8 expression, recombination human IL-8 (rhIL-8) was added to HNSCC cell lines, and a series of experiments was performed in vitro. The wound healing assay showed that the migration ability of HN4, HN6, and Cal27 increased relative to the concentration of rhIL-8 (Fig. 2a, *P* < 0.01). The transwell assays indicated that the number of cells in the lower chambers increased relative to rhIL-8 concentration with or without Matrigel (Fig. 2b, c, *P* < 0.01). The colony-forming assay had a similar result: the group with 10 ng/ml and 100 ng/ml rhIL-8 had an incremental increase in number of clone clusters (Fig. 2d, *P* < 0.01). Cell proliferation was also significantly enhanced by rhIL-8 (Fig. 2e, *P* < 0.05). Western blot analysis revealed a decrease in the expression of E-cadherin and an increase in the expression of MMP2, MMP9, and vimentin in the rhIL-8 treatment group (Fig. 2f, Supplementary Fig. 1a–d). These results suggest that IL-8 enhanced the capacity of metastasis and proliferation in HNSCC cells.

**Fig. 3:**
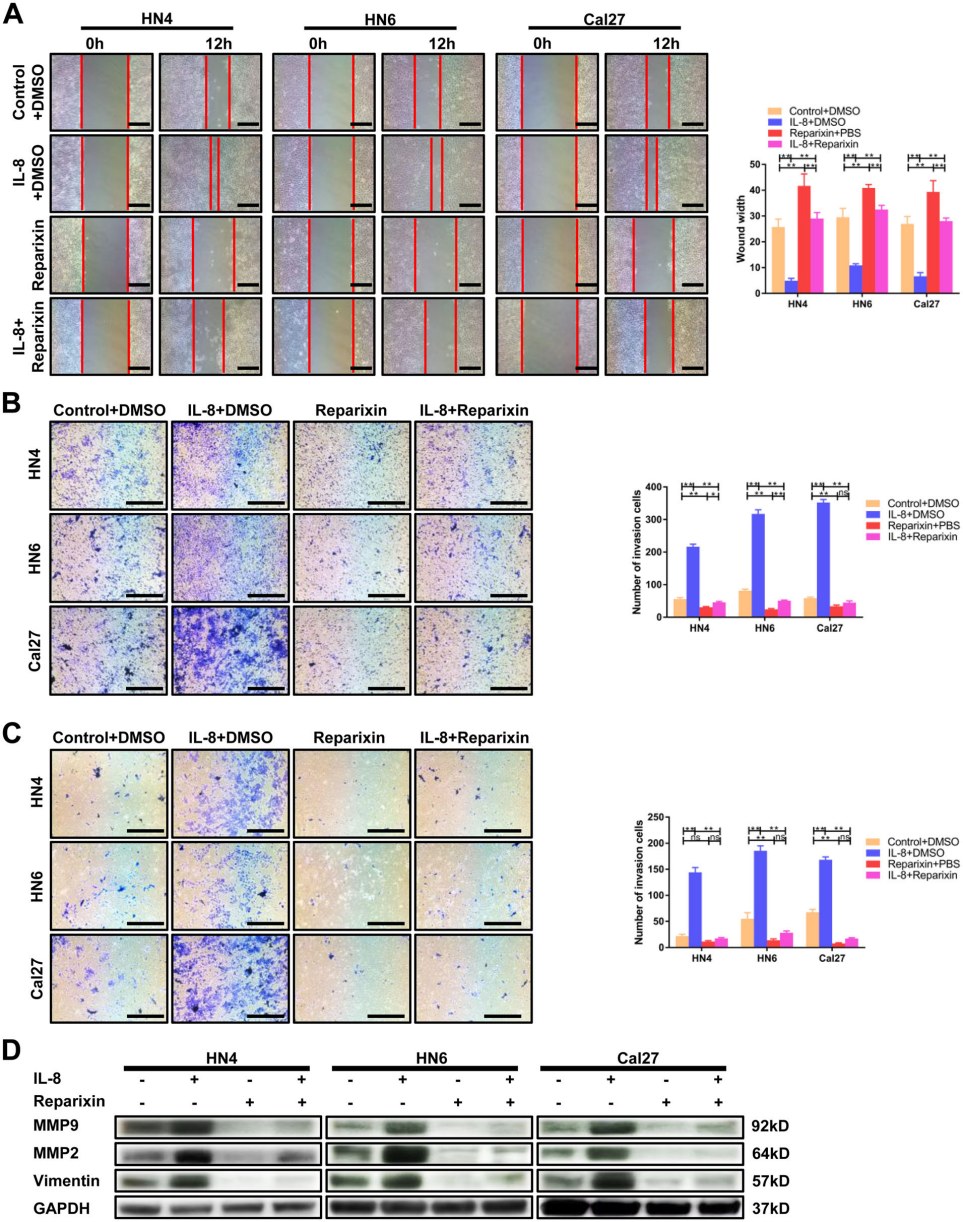
A CXCR1/2 blocker inhibits IL-8-induced cancer-promoting activity. **a** A wound healing assay was performed to detect the migration ability of HSNCC cells treated with 100 ng/ml IL-8 or 50 μg/ml Reparixin as the indication shows. Scale bar, 100 μm. **b**, **c** Transwell assays were performed to determine the migration and invasion ability of cells with the indicated stimulation. Scale bar, 250 μm. **d** MMP9, MMP2, and vimentin were detected after stimulation with 100 ng/ml IL-8 and 50 μg/ml Reparixin using western blot; *P < 0.05, **P < 0.01.

It has been reported that CXCR1/2 are the receptors for IL-8 on tumor cells. Survival assay confirmed that high concentration of Reparixin, a CXCR1/2 blocker, could significantly affect the survival of HNSCC cells (Supplementary Fig. 2a). In this case, we selected 50 μg/ml as a proper concentration for follow-up experiments to maximize the block effect of CXCR1/2 with the minimal cytotoxicity. The results of the wound healing assay, migration assay, and invasion assay showed that Reparixin could notably reverse the promotion effect mediated by rhIL-8 stimulation (Fig. 3a–c). Western blot also revealed that the increased expression of MMP2, MMP9, and vimentin was blocked by Reparixin (Fig. 3d, Supplementary Fig. 3a–c). Interestingly, we found that using Reparixin alone could also inhibit the migration and invasion ability of HNSCC cells. These results suggested that Reparixin may block both the exogenous IL-8 and autocrine IL-8 of tumor cells.

### IL-8 facilitates HNSCC progression in vivo through CXCR1/2

**Fig. 4:**
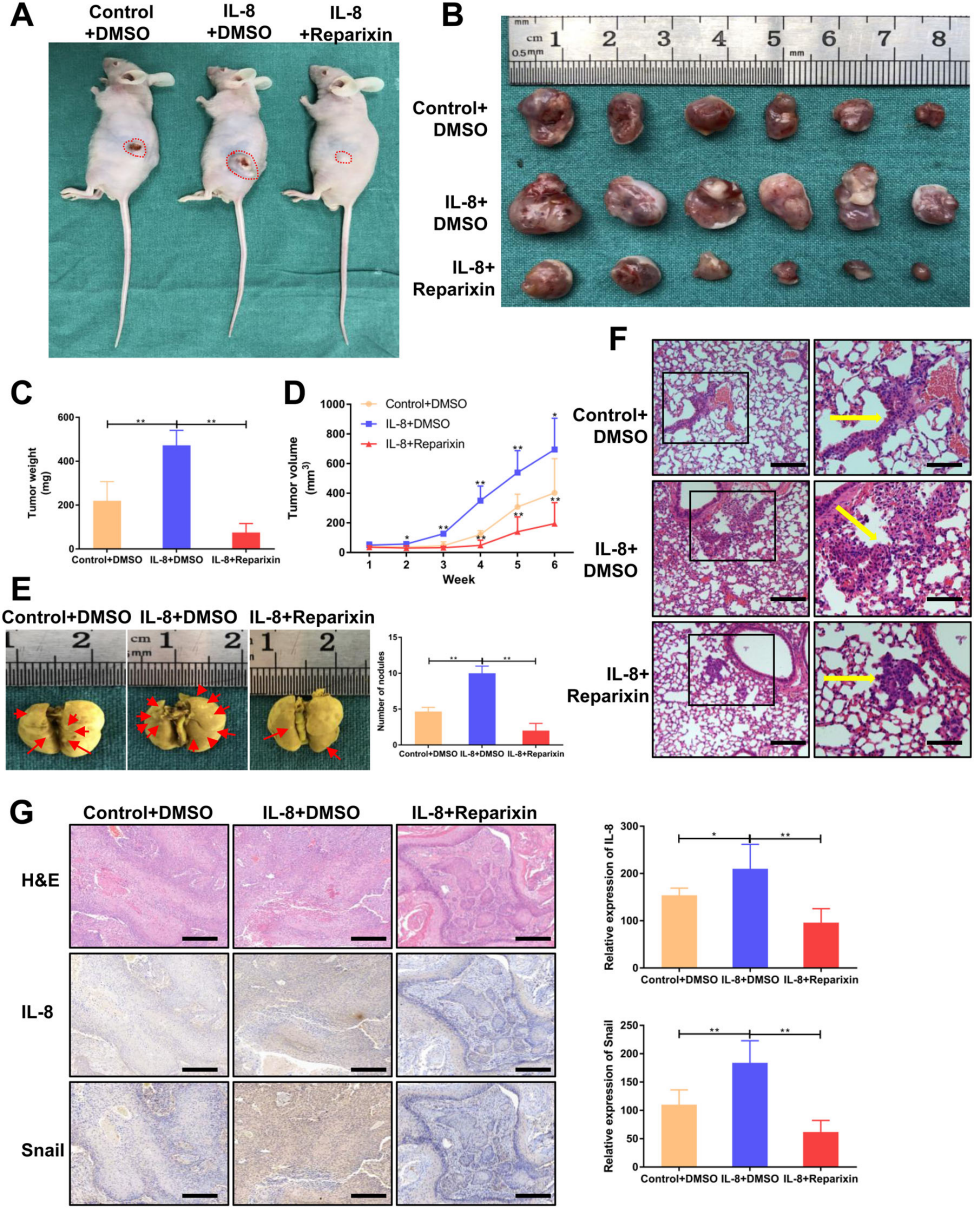
IL-8 facilitates HNSCC progression in vivo through CXCR1/2. **a**, **b** Representative images of xenograft tumors derived from mice treated with IL-8 (0.5 mg/kg, every other day) + DMSO, IL-8 (0.5 mg/kg, every other day) + Reparixin (15 mg/kg, once a day), or PBS + DMSO; (*n* = 6). **c** Tumor weight was measured after excision from mice in three groups. **d** Tumor volume was examined every week and plotted as tumor growth curves. IL-8 + DMSO group and IL-8 + Reparixin group were compared with the control group. **e** Representative lung tissues from different groups in the metastasis assay were fixed in Bouin’s fixative diluted 1:5 with neutral-buffered formalin; (*n* = 6). Arrows indicate metastatic nodules. **f** H&E staining of lung tissues was performed to examine the pathologic type of the metastatic nodules (magnification 100×, 200×). Scale bar, 100 μm and 50 μm. Arrows indicate metastatic cell nests. **g** H&E and IHC staining for IL-8, and snail were conducted on xenograft tumors (magnification 100×). Scale bar, 100 μm; *P < 0.05, **P < 0.01.

To confirm the results of the in vitro studies, rhIL-8 and Reparixin were injected into mice as described. Similar to the in vitro study, tumors treated with rhIL-8 grew fastest in all three groups, while the IL-8 + Reparixin group grew slowest (Fig. 4a–c). This trend started at week 2 and can be observed throughout the whole process (Fig. 4d). Tumor growth could also be limited by using Reparixin alone (Supplementary Fig. 2b–e). In addition to its proliferation ability, the migration and invasion abilities promoted by IL-8 were determined by using a lung metastasis model. The results showed that IL-8 significantly increased the number of metastatic nodules in the lung, which was inhibited by the CXCR1/2 blocker (Fig. 4e). H&E staining was performed to identify the pathologic type of metastasis nodules (Fig. 4f). In accordance with our previous results, IHC staining for xenograft tumors showed that IL-8 and snail expression were significantly higher in the IL-8 treatment group than in the control group (Fig. 4g). This result implied that IL-8 can initiate the EMT program to promote the malignant progression of HNSCC.

### The phosphorylation of STAT3 is key downstream of IL-8

**Fig. 5:**
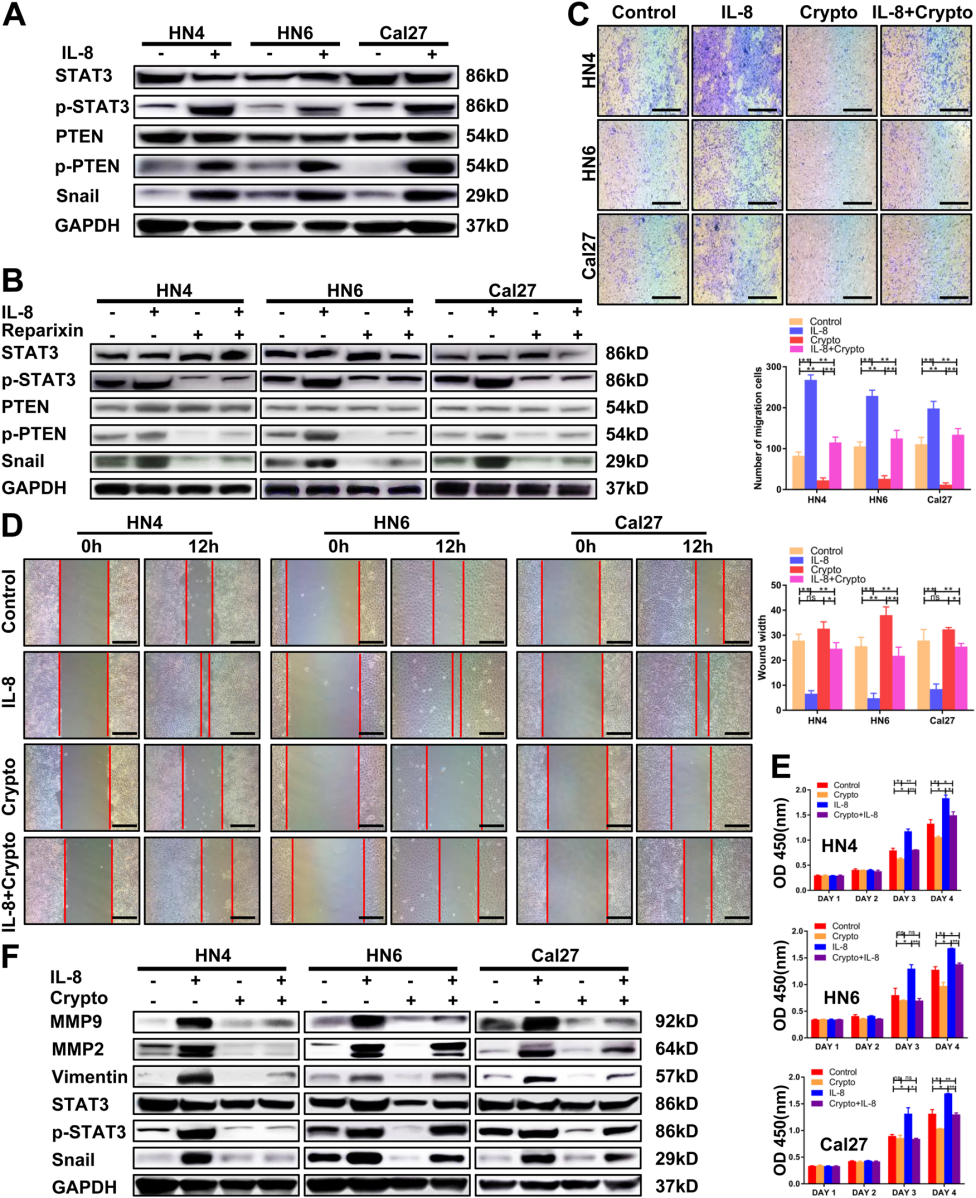
IL-8 promotes HNSCC progression through the STAT3 pathway. **a** The expression of STAT3, p-STAT3, PTEN, p-PTEN, and snail was detected by western blot after 100 ng/ml IL-8 treatment. **b** STAT3, p-STAT3, PTEN, p-PTEN, and snail expression were determined by western blot after 100 ng/ml IL-8 and 50 μg/ml Reparixin treatment. **c**, **d** Migration ability was examined by a transwell assay and a wound healing assay after stimulation with 100 ng/ml IL-8, 10 μM crypto, 100 ng/ml IL-8 + 10 μM crypto, or blank medium. Scale bar, 250 μm (transwell assay) and 100 μm (wound healing assay). **e** The CCK8 assay was applied to detect proliferation ability. **f** The expression of MMP9, MMP2, vimentin, STAT3, p-STAT3, and snail in each group was detected by western blot; *P < 0.05, **P < 0.01.

As a critical oncogene, STAT3, has been proven to be associated with malignant tumor progression, through processes such as proliferation, migration, and invasion. Our study found that a high concentration of IL-8 induced the phosphorylation of STAT3 and then increased the expression of snail (Fig. 5a, Supplementary Fig. 4a, b). This process could be blocked by Reparixin for both rhIL-8 and endogenous IL-8 (Fig. 5b, Supplementary Fig. 4d, e). To further prove the function of STAT3 in this process, cryptotanshinone (crypto), an inhibitor of the phosphorylation of STAT3, was applied in migration and proliferation experiments at 10 μM based on our previous research. The migration and proliferation abilities mediated by IL-8 were significantly inhibited by crypto (Fig. 5c–e). Western blot also showed that the expression of MMP2, MMP9, vimentin, and snail were decreased along with p-STAT3 inhibition (Fig. 5f, Supplementary Fig. 4g–k).

Interestingly, we found that the phosphorylation of PTEN was also increased by the stimulation of IL-8 and could be blocked by Reparixin, which implied that PTEN was also regulated by IL-8 (Fig. 5a, b, Supplementary Fig. 4c, f). Therefore, we assumed that PTEN must also be key in the downstream signaling of IL-8 in HNSCC. However, the relationship between PTEN and p-STAT3 requires further investigation.

### The inactivation of PTEN plays a pivotal role in STAT3 activation and IL-8 secretion

**Fig. 6:**
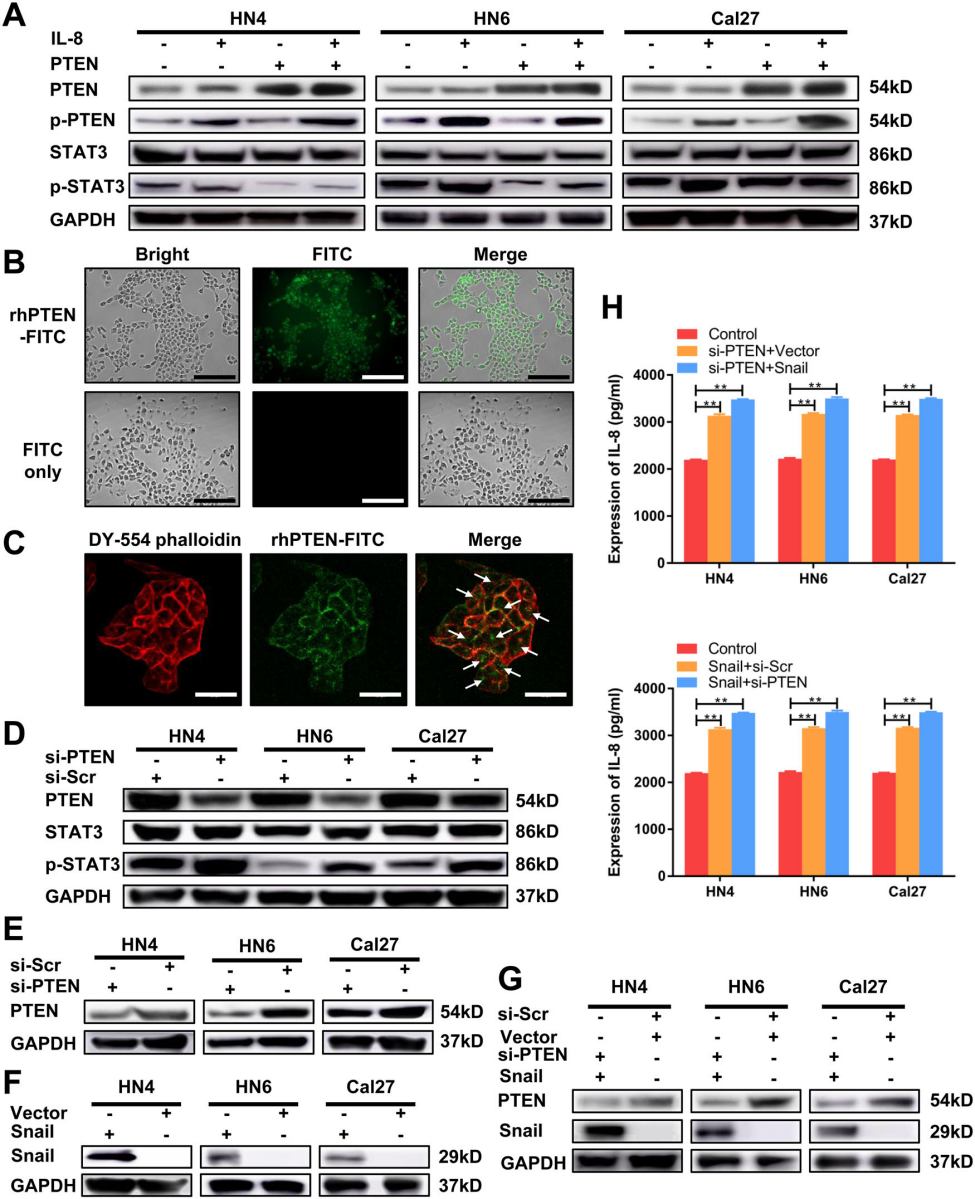
The inactivation of PTEN plays a pivotal role in STAT3 activation and IL-8 secretion. **a** The expression levels of PTEN, p-PTEN, STAT3, and p-STAT3 were examined after 100 ng/ml IL-8 and/or PTEN stimulation. **b** The fluorescent images of Cal27 cells treated with rhPTEN-FITC or FITC alone for 24 h. Scale bar, 200 μm. **c** Cal27 cells incubated with rhPTEN-FITC and DY-554 phalloidin were detected by confocal immunofluorescent analysis. White arrows indicate the rhPTEN-FITC internalized by HNSCC cells. Scale bar, 50 μm. **d** PTEN, STAT3 and p-STAT3 expression was detected by western blot after transfection with si-PTEN or si-scramble. **e**–**g** The transfection efficiency was validated by western blot. **h** ELISA was performed to detect the secretion of IL-8 in HNSCC cells after transfection with si-PTEN and/or snail vector; **P < 0.01.

As a form of inactivation, the phosphorylation of PTEN loses its ability to inhibit tumors. Our study proves that IL-8 promotes the inactivation of PTEN and the activation of STAT3. Recombinant PTEN (rhPTEN) was applied to explore the relationship between PTEN and p-STAT3. We observed that the expression of p-STAT3 in the combination group was markedly suppressed compared to that in the rhIL-8 group (Fig. 6a, Supplementary Fig. 5a–c). The fluorescent images showed that fluorescein isothiocyanate (FITC)-labeled rhPTEN could be observed inside HNSCC cells (Fig. 6b, Supplementary Fig. 6). Furthermore, confocal immunofluorescent analysis of HNSCC cells obviously demonstrated that rhPTEN protein could be internalized into cytoplasm (Fig. 6c). The results above not only proved that the exogenous PTEN ingested by HNSCC cells inhibited the activation of STAT3 by IL-8, but also suggested that rhPTEN was able to be internalized by HNSCC cells. To further prove this point, plasmids containing PTEN were transfected into cells. The results of western blot showed that with the increase of PTEN level, p-STAT3 level could not be upregulated even treated with IL-8, which was consist with the finding above (Supplementary Fig. 7a, b). Apparently, exogenous PTEN weakened IL-8-induced STAT3 activation to decrease STAT3 phosphorylation. Conversely, knockdown of PTEN expression significantly increased the p-STAT3 expression (Fig. 6d, Supplementary Fig. 5d, e). These results demonstrated that PTEN was an important switch in the IL-8/STAT3 pathway.

To further investigate whether these downstream molecules can affect IL-8 production, we cotransfected small interfering RNA (siRNA) targeting PTEN and plasmids targeting snail into HNSCC cells, and analyzed the supernatant medium. The transfection efficiency was validated by western blot (Fig. 6e–g, Supplementary Fig. 5f–i). ELISA showed that the secretion of IL-8 was significantly higher in the si-PTEN or snail group than in the control group (Fig. 6h). Moreover, the cotransfection group had markedly increased the secretion of IL-8 compared to that in the single-transfection group (Fig. 6h). The results above proved that there was a synergistic effect between PTEN knockdown and snail upregulation in the secretion of IL-8 on HNSCC cells.

### IL-8 expression positively correlates with snail and vimentin expression in HNSCC tissues

**Fig. 7:**
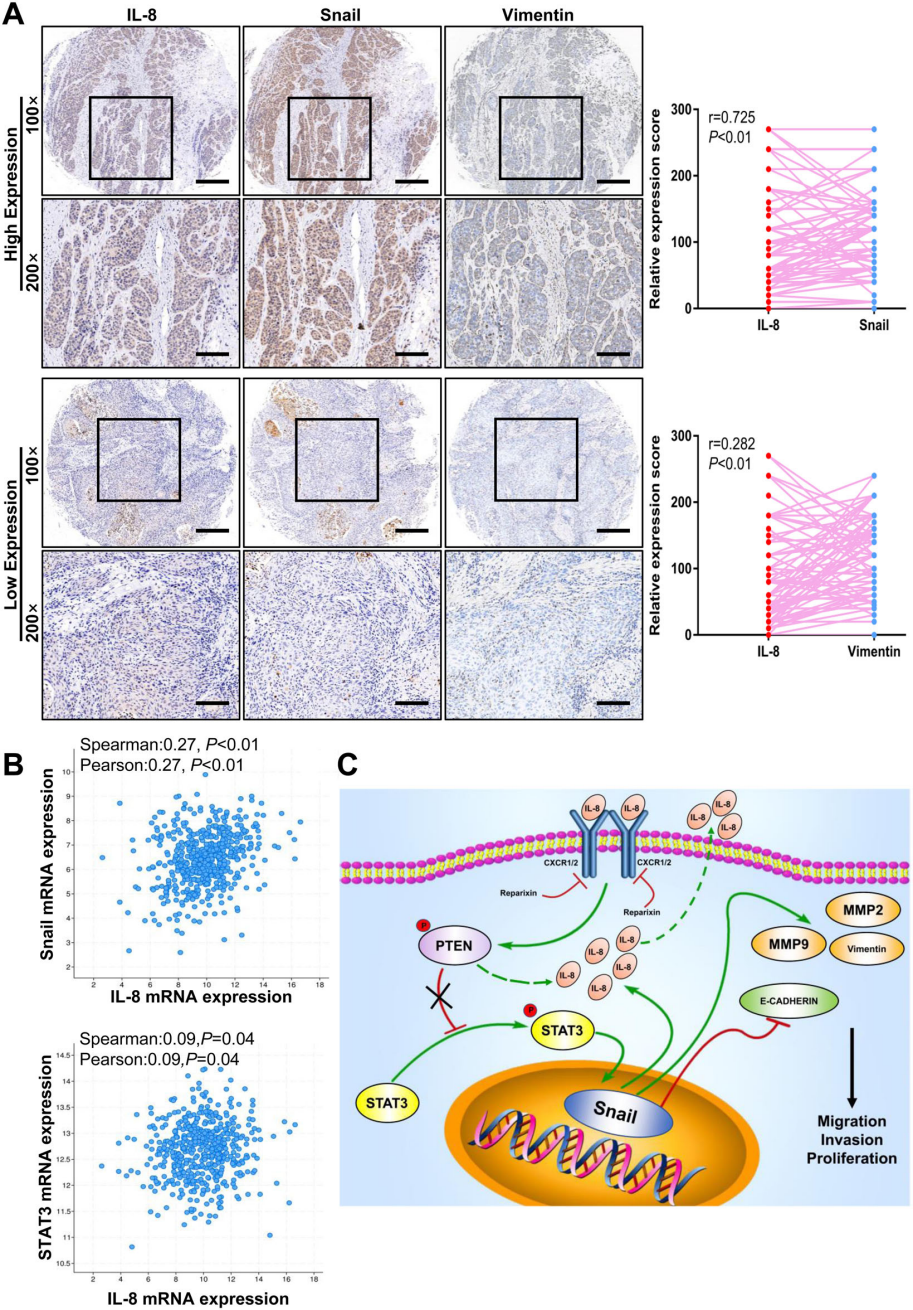
IL-8 expression positively correlates with snail and vimentin in HNSCC tissues. **a** Representative IHC images and correlation analysis of IL-8, snail, and vimentin from 84 HNSCC tumor tissues (magnification 100×, 200×) are presented. Scale bar, 100 μm and 50 μm. **b** The relationship between IL-8, snail, and STAT3 was analyzed based on the TCGA dataset. **c** A schematic diagram shows that IL-8 phosphorylates PTEN to activate the STAT3/snail/EMT process and creates an IL-8/PTEN/snail positive feedback loop, which plays a critical role in HNSCC progression; *P < 0.05, **P < 0.01.

Based on the above results, we concluded that IL-8 can notably promote the expression of invasion-associated molecules. This finding was further confirmed by IHC staining of patients’ tissue samples. Eighty-four tumor tissues were used to detect the expression of IL-8, snail, and vimentin. The results indicated that the expression level of IL-8 was remarkably related to snail (Fig. 7a, *r* = 0.725, *P* < 0.01) and vimentin (Fig. 7a, *r* = 0.282, *P* < 0.01). Correlation analysis of the TCGA dataset also confirmed the positive correlation between IL-8, snail, and STAT3 in the HNSCC dataset (Fig. 7b).

These data reveal that IL-8 binds to CXCR1/2 on HNSCC cells and then phosphorylates PTEN to activate STAT3. p-STAT3 enters the nucleus to enhance the expression of snail, which leads to the expression of EMT-related proteins. This process is manifested as cell migration, invasion, and proliferation, which cause the poor outcomes of HNSCC patients (Fig. 7c).

## Discussion

As one of the most important causes of poor prognosis, there are an increasing number of studies concerning tumor metastasis in HNSCC (refs. ^22,23^). This study demonstrates that the interaction between IL-8 and PTEN inactivation plays a critical role in this process through STAT3 activation. Moreover, the inactivation of PTEN has a strong impact on the phosphorylation of STAT3, which indicates that PTEN is a novel molecular switch of STAT3 signaling.

IL-8, a member of interleukin family, has been discovered for many years, and its oncogenic function has been reported in many studies^24,25^. In this study, we demonstrated that HNSCC patients have significantly higher expression of IL-8 than normal counterparts. The level of IL-8 is related to aggressive clinical characteristics and outcome. In accordance with previous papers, we showed the function of IL-8 in promoting malignant progression in HNSCC. This process is mediated by CXCR1/2, the receptors of IL-8 on tumor cells. Huang et al. claimed that the activation of CXCR1 promoted the invasive and metastatic abilities of hepatocellular carcinoma cells^26^. In our study, we found that Reparixin could not only block the effect of rhIL-8, but also inhibit the proliferation and progression after administration alone. These findings further proved that tumor cells were able to secrete IL-8, and it’s critical for the occurrence and development of HNSCC. At present, the mainstream academic view is that tumor cells secrete IL-8 under environmental pressure. In this study, we prove that the inactivation of PTEN can also enhance the secretion of IL-8, which creates a positive feedback loop.

The role of PTEN in tumor suppression has been demonstrated in many cancers^27,28^. The deficiency and inactivation of PTEN results in the loss of its limiting effect on many cancer-promoting factors^29^. Nevertheless, the relationship between PTEN and STAT3 is lacking. In this study, we confirmed that IL-8 promotes the phosphorylation and inactivation of PTEN. Current studies have discovered that PTEN removes the phosphate group from PIP3 and transforms it to PIP2, which inhibits the activation of the AKT pathway^30,31^. Our research found that the phosphorylation level of STAT3 increased significantly when tumor cells lost PTEN and decreased when PTEN levels increased. In these experiments, we demonstrated that the inhibition of STAT3 activation was a new biological function of PTEN during carcinogenesis. Moreover, due to its significant effect related with tumor occurrence and development, the restoration of PTEN level is also an important topic in many cancers^32^. Our study firstly demonstrated that HNSCC cells are able to ingest exogenous PTEN protein that provide a brand-new method for this topic. However, the underlying mechanism of how this extracellular protein get into HNSCC cells is still unknown and need further exploration.

The functions of STAT3 in promoting tumor invasion and migration have been explored in many cancers, including HNSCC (refs. ^33,34^). Our experiments prove that p-STAT3 enhances the level of snail, which facilitates EMT by regulating the expression of E-cadherin, MMP2, MMP9, and vimentin. Interestingly, the upregulation of snail can, in turn, promote IL-8 secretion. In this study, IL-8, PTEN, and snail form a positive feedback loop. IL-8 phosphorylates PTEN to activate snail and then inactivates PTEN, and ectopic expression of snail increases the secretion of IL-8, which further stimulates the PTEN inactivation and snail expression. This loop can make a great contribution to tumor metastasis. However, there are still some unsolved problems that require future exploration. First, whether the relationship between PTEN and STAT3 is direct requires gene-level regulation for further verification. Second, whether PTEN promotes IL-8 levels directly or through increased expression of snail remains obscure. Finally, the role of IL-8 as an immune-related chemokine in this process is worth discussion. These questions will be further explored in our future study.

In summary, this study demonstrates that IL-8 acts as a critical oncogene, and that the interaction of IL-8 and PTEN promotes HNSCC progression via STAT3 signaling by facilitating the EMT program. Moreover, the crosstalk between PTEN and STAT3 provides new insight into the tumor development mechanism, which has great significance for the exploration of HNSCC progression and new therapeutic targets.

## Materials and methods

### Patient and tissue samples

A total of 106 patients from November 2006 to April 2011, including primary and recurrent HNSCC were enrolled in this study, and 111 normal volunteers were enrolled for comparison. Moreover, 84 HNSCC tissue samples from January 2007 to December 2008 were collected to be analyzed by immunohistochemistry staining. All patients enrolled have signed the informed consent, and were pathologically diagnosed with squamous cell carcinoma and had complete clinicopathological data. The tissue samples and medical records of patients enrolled were collected via strict procedures. This study was conducted in full accordance with ethical principles and approved by the Medical Ethics Committee of the Ninth People’s Hospital, Shanghai Jiao Tong University, School of Medicine.

### Cell culture

Cal27, HN4, HN6, HN30, SCC4 cell lines, along with the HIOEC line, were used in this study. All cell lines were recently authenticated by STR profiling and no mycoplasma contamination was detected. SCC4 and Cal27 were purchased from ATCC (Manassas, VA). The cell lines HN4, HN6, and HN30 were kindly provided by the University of Maryland Dental School, USA. All these cell lines were cultured in Dulbecco’s modified Eagle’s medium (Gibco, Carlsbad, CA) supplemented with 10% fetal bovine serum, 1% glutamine, and 1% penicillin–streptomycin. rhIL-8 (Peprotech, USA) and recombinant human PTEN (847-PN, R&D, USA) were added to the medium to function as exogenous stimulation. Cells were cultured in a standard humidified atmosphere of 5% CO_2_ at 37 °C.

### Immunohistochemistry

Immunohistochemical (IHC) staining was performed to detect IL-8, snail, and vimentin expression in HNSCC patient tissue samples. After deparaffinization and rehydration, the tissue slides were heated in a water bath at 100 °C with citrate buffer solution (pH 6.0) for 20 min to retrieve antigen, and then cooled at room temperature. The primary antibodies were incubated overnight at 4 °C in a humidified chamber and then visualized using a 3,3′-diaminobenzidine (DAB) detection kit (Dako Cytomation, Denmark) containing goat secondary antibody molecules and DAB chromogen. Every step of the wash used phosphate-buffered saline solution (PBS) for 5 min repeated three times. The primary antibodies (with their dilutions reported), including IL-8 (1:1000; ab18672, Abcam, USA), snail (1:1000; ab53519, Abcam, USA), and vimentin (1:200; D21H3, Cell Signaling Technology, USA) were used as the manufacturer’s instructions suggest. The intensity of the IL-8, snail, and vimentin immunoreaction was scored as follows: 0 = negative, absence of stained cells; 1 = weak; 2 = moderate; and 3 = strong. The IHC staining score was calculated by multiplying the percentage of positive cells by the staining intensity. The scoring was conducted by researchers who were blind to the clinical information of patients.

### Cell transfection

Plasmids encoding snail or PTEN and small interfering RNA (siRNA) for PTEN (Genomeditech, Shanghai, China), as well as empty vector and scramble sequence were transiently transfected into HN4, HN6, and Cal27 cells using Lipofectamine™ 3000 (Invitrogen, Carlsbad, CA, USA) according to the manufacturer’s instructions. The subsequent treatments and experiments were performed 24 h after transfection. The sequences of the PTEN siRNA were as follows: #1, 5′-CCACAAAUGAAGGGAUAUAAATT-3′; #2, 5′-CUAGAACUUAUCAAACCCUUUTT-3′; and #3, 5′-GUAUAGAGCGUGCAGAUAATT-3′. siRNA #1 was chosen because it had the highest silencing efficiency.

### Cellular proliferation, migration, and invasion assay

Cell counting kit (CCK8; Dojindo, Kumamoto, Japan) assays were used to detect cell proliferation. A total of 1 × 10^3^ cells per well were plated into six-well plates for colony-forming assays and were fixed and stained after 10–14 days. Migration ability was examined by both a wound healing assay and a transwell assay (uncoated insert), as we described in our previous study^35^. A transwell assay (Matrigel-coated insert) was used for invasion monitoring. The migration and invasion assays were all performed in a serum-free environment to avoid the influence of proliferation. All experiments were performed three times, and the cell numbers were counted at least three times to calculate an average.

### Real-time PCR

To test the expression level of IL-8, RT-PCR was performed using tissue samples collected from HNSCC patients and normal volunteers. RT-PCR was performed according to the manufacturer’s instructions and as described in our previous study^36^. The primer sequences were as follows: IL-8 forward: 5′-GTGCAGTTTTGCCAAGGAGT-3′ and reverse: 5′-ATGAATTCTCAGCCCTCTTCAA-3’; and GAPDH forward: 5′-CCTCTGACTTCAACAGCGAC-3′ and reverse: 5′-TCCTCTTGTGCTCTTGCTGG-3′. Melt curve was used for the validation of the primers.

### Enzyme-linked immunosorbent assay

The IL-8 content in the cell supernatant was detected by using an ELISA kit (D8000C, R&D, USA). The cell supernatant was collected after culture for 24 h and measured following the manufacturer’s instructions. The optical density of each well was calculated by reading the wavelength of 450 nm and then subtracting the reading at 540 nm. The concentration of IL-8 was converted from the standard curve.

### Protein FITC labeling and imaging

The rhPTEN was labeled using FITC isomer I labeling kit (786-141, G-Biosciences, USA). The protein was incubated with labeling agent for 1 h at room temperature and centrifuged in a filter column. The labeled rhPTEN was then applied into cell medium and treated for 24 h. Cell medium was washed thoroughly by PBS and then fixed by paraformaldehyde for 15 min before DY-554 phalloidin applied. For imaging analysis, the rhPTEN-FITC and bright field were detected by fluorescence microscope. The rhPTEN-FITC and DY-554 phalloidin were detected by confocal laser microscope. The image of cell in bright field or RFP field was merged with the GFP field.

### Western blot analysis

Western blotting was performed as previously described^37^. The antibodies used in this study were E-cadherin (24E10, Cell Signaling Technology, USA), MMP9 (D6O3H, Cell Signaling Technology, USA), MMP2 (D4M2N Cell Signaling Technology, USA), vimentin (D21H3, Cell Signaling Technology, USA), PTEN (D4.3, Cell Signaling Technology, USA), p-PTEN (44A7, Cell Signaling Technology, USA), STAT3 (D3Z2G, Cell Signaling Technology, USA), p-STAT3 (D3A7, Cell Signaling Technology, USA), snail (C15D3, Cell Signaling Technology, USA), and GAPDH (D16H11, Cell Signaling Technology, USA). GAPDH was used as an internal control. The immunoreactive bands were visualized with ECL Ultra (New Cell and Molecular Biotech, Suzhou, China). All western blots were repeated three times with separate cell lysates and the statistical analysis were provided in the supplementary figures.

### Animal studies

Six-week-old BALB/c male nude mice purchased from the Shanghai Laboratory Animal Center (Shanghai, China) were bred in SPF facilities. The mice were randomly divided into each group (*n* = 6) and the animal study was conducted by the researchers, who were blind to the treatment. A total of 1 × 10^6^ Cal27 cells were injected into the left or right flanks of mice for tumorigenicity evaluation. For the lung metastasis evaluation, 1 × 10^6^ Cal27 cells were injected through the lateral tail vein in separate mice. rhIL-8 (0.5 mg/kg, s.c., every other day) and Reparixin (Abmole, USA, 15 mg/kg, i.p., once a day) were strictly administered; PBS and DMSO, respectively, were the solvent controls. Tumor volume was measured once a week. The weight and volume of the tumors were finally measured after the mice were sacrificed at the end point. Lung tissues were removed and fixed in Bouin’s fixative diluted 1:5 with neutral-buffered formalin for nodule observation^38^. Both lung and tumor tissues were fixed and stained before microscopic analysis. The in vivo studies were approved by the Animal Care and Use Committee of Ninth People’s Hospital, Shanghai Jiao Tong University School of Medicine.

### Statistical analysis

The data were analyzed by SPSS 13.0 for Windows (SPSS Inc., Chicago, IL) and plotted by GraphPad Prism version 6 (GraphPad Software, San Diego, CA, USA). Student’s *t*-test and one-way ANOVA were performed to assess the statistical significance of differences. Survival analysis was conducted using the Kaplan–Meier method and log-rank test. *P* < 0.05 was considered statistically significant (*P < 0.05, and **P < 0.01). All values are expressed as the means ± standard errors.

## Supplementary information

Supplementary Figure Legends

Supplementary Figure 1

Supplementary Figure 2

Supplementary Figure 3

Supplementary Figure 4

Supplementary Figure 5

Supplementary Figure 6

Supplementary Figure 7
