# Supplementary material for: The interaction of interleukin-8 and PTEN inactivation promotes the malignant progression of head and neck squamous cell carcinoma via the STAT3 pathway
Source: Cell Death Dis. 2020 May 29;11(5):405. doi: 10.1038/s41419-020-2627-5 (PMC7260373; doi:10.1038/s41419-020-2627-5)
Supplement: Supplementary file 1 — Supplementary Figure Legends [file 41419_2020_2627_MOESM1_ESM.docx]

**Supplementary Figure Legends**

**Fig. S1. Statistical analysis of Western blot in Figure 2.** The statistical analysis of the expression of E-cadherin **a**, MMP9 **b**, MMP2 **c** and vimentin **d** after treated with IL-8. **P* < 0.05, ***P* < 0.01.

**Fig. S2 Reparixin has an anti-tumor effect for HNSCC. a** Survival rate was determined by CCK8 assay treated with different concentration of Reparixin. **b, c** Representative images of xenograft tumors derived from mice treated with Reparixin (15 mg/kg, once a day) or DMSO. (n=6) **d** Tumor volume was measured every week and plotted as tumor growth curves. **e** Tumor weight was measured after excision from mice in both groups. **P* < 0.05, ***P* < 0.01.

**Fig. S3. Statistical analysis of Western blot in Figure 3.** The statistical analysis of the expression of MMP9 **a**, MMP2 **b** and vimentin **c** after treated with IL-8 and Reparixin. **P* < 0.05, ***P* < 0.01.

**Fig. S4. Statistical analysis of Western blot in Figure 5.** The statistical analysis of the expression of p-STAT3/STAT3 **a**, snail **b** and p-PTEN/PTEN **c** after treated with IL-8. The expression of p-STAT3/STAT3 **d**, snail **e** and p-PTEN/PTEN **f** were analyzed after treated with IL-8 and Reparixin. MMP9 **g**, MMP2 **h** and vimentin **i**, p-STAT3/STAT3 **j** and snail **k** were detected and analyzed after IL-8 and crypto were applied. **P* < 0.05, ***P* < 0.01.

**Fig. S5. Statistical analysis of Western blot in Figure 6.** The statistical analysis of the expression of PTEN **a**, p-PTEN/PTEN **b** and p-STAT3/STAT3 **c** after IL-8 and PTEN were applied. The expression of PTEN **d** and p-STAT3/STAT3 **e** were analyzed after transfected si-PTEN. The transfected efficiency of si-PTEN or snail were analyzed for the expression of PTEN **f** and snail **g**. PTEN **h** and snail **i** were also detected and analyzed after transfected with both si-PTEN and snail. **P* < 0.05, ***P* < 0.01.

**Fig. S6. Fluorescent images of HN4 and HN6 cells treated with rhPTEN-FITC or FITC alone.** Scale bar, 200 μm.

**Fig. S7. PTEN inhibits the phosphorylation of STAT3. a** PTEN, p-PTEN, STAT3 and p-STAT3 were detected by Western blot after transfected with PTEN or vector plasmids. **b** The statistical analysis of the expression of PTEN, p-PTEN/PTEN and p-STAT3/STAT3 after PTEN or vector plasmids were transfected.
