# Supplementary figures and images for: The interaction of interleukin-8 and PTEN inactivation promotes the malignant progression of head and neck squamous cell carcinoma via the STAT3 pathway
Source: Cell Death Dis. 2020 May 29;11(5):405. doi: 10.1038/s41419-020-2627-5 (PMC7260373; doi:10.1038/s41419-020-2627-5)

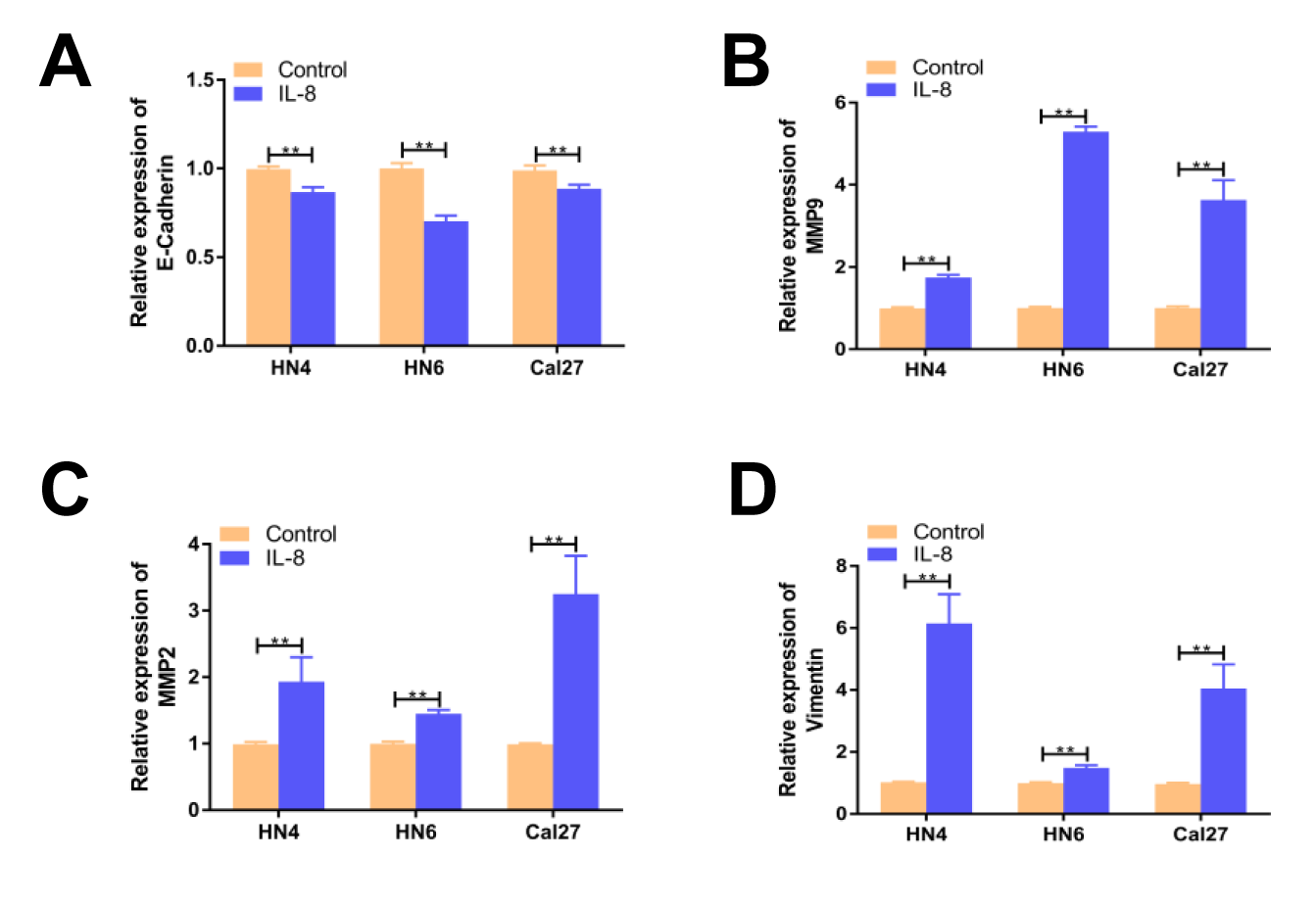

Supplement: Supplementary file 2 — Supplementary Figure 1 [file 41419_2020_2627_MOESM2_ESM.tif]

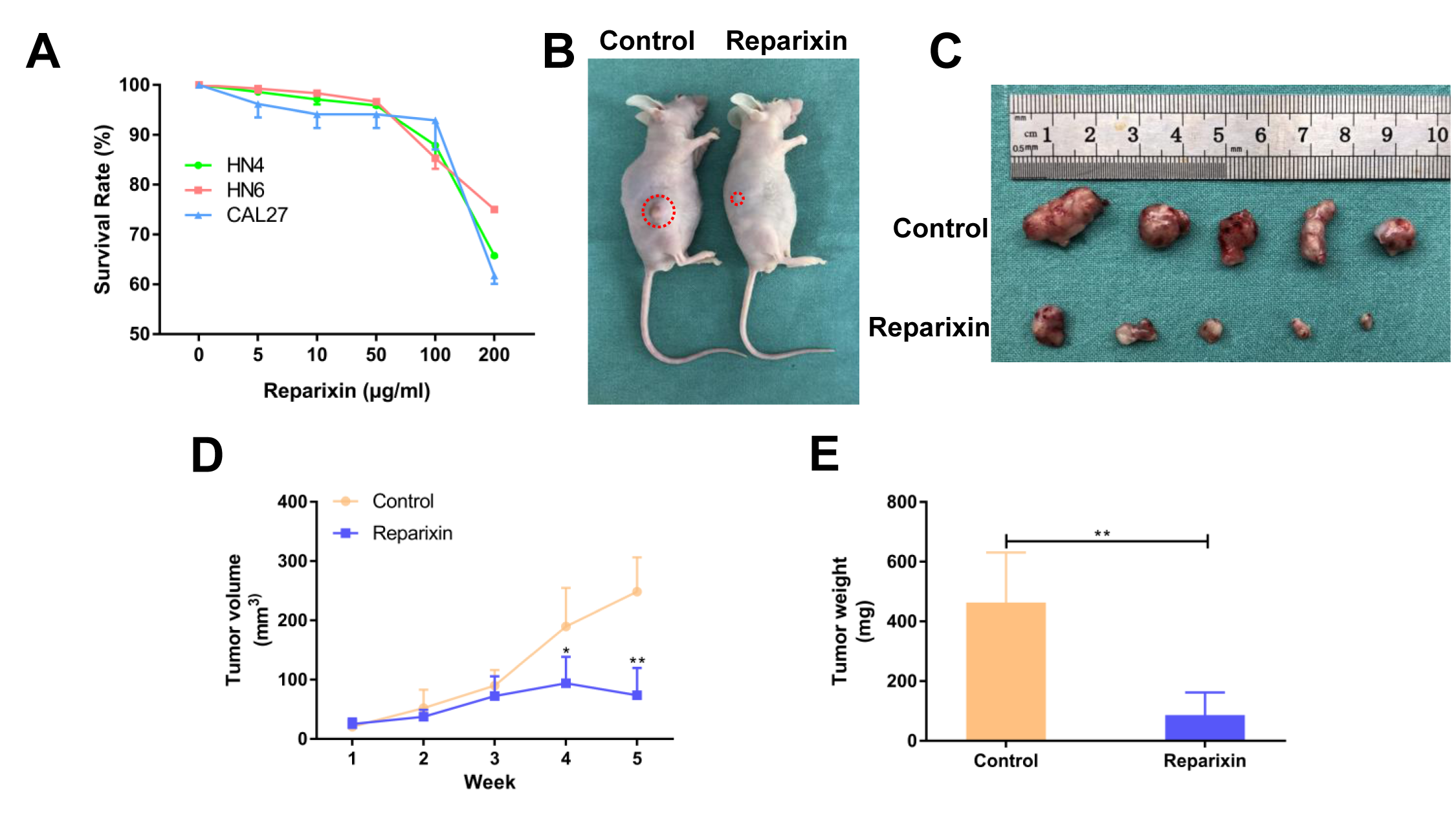

Supplement: Supplementary file 3 — Supplementary Figure 2 [file 41419_2020_2627_MOESM3_ESM.tif]

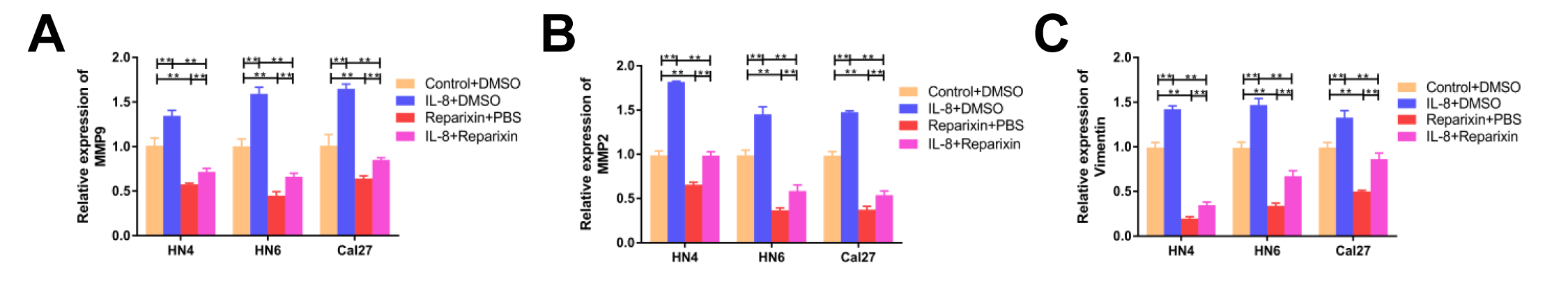

Supplement: Supplementary file 4 — Supplementary Figure 3 [file 41419_2020_2627_MOESM4_ESM.tif]

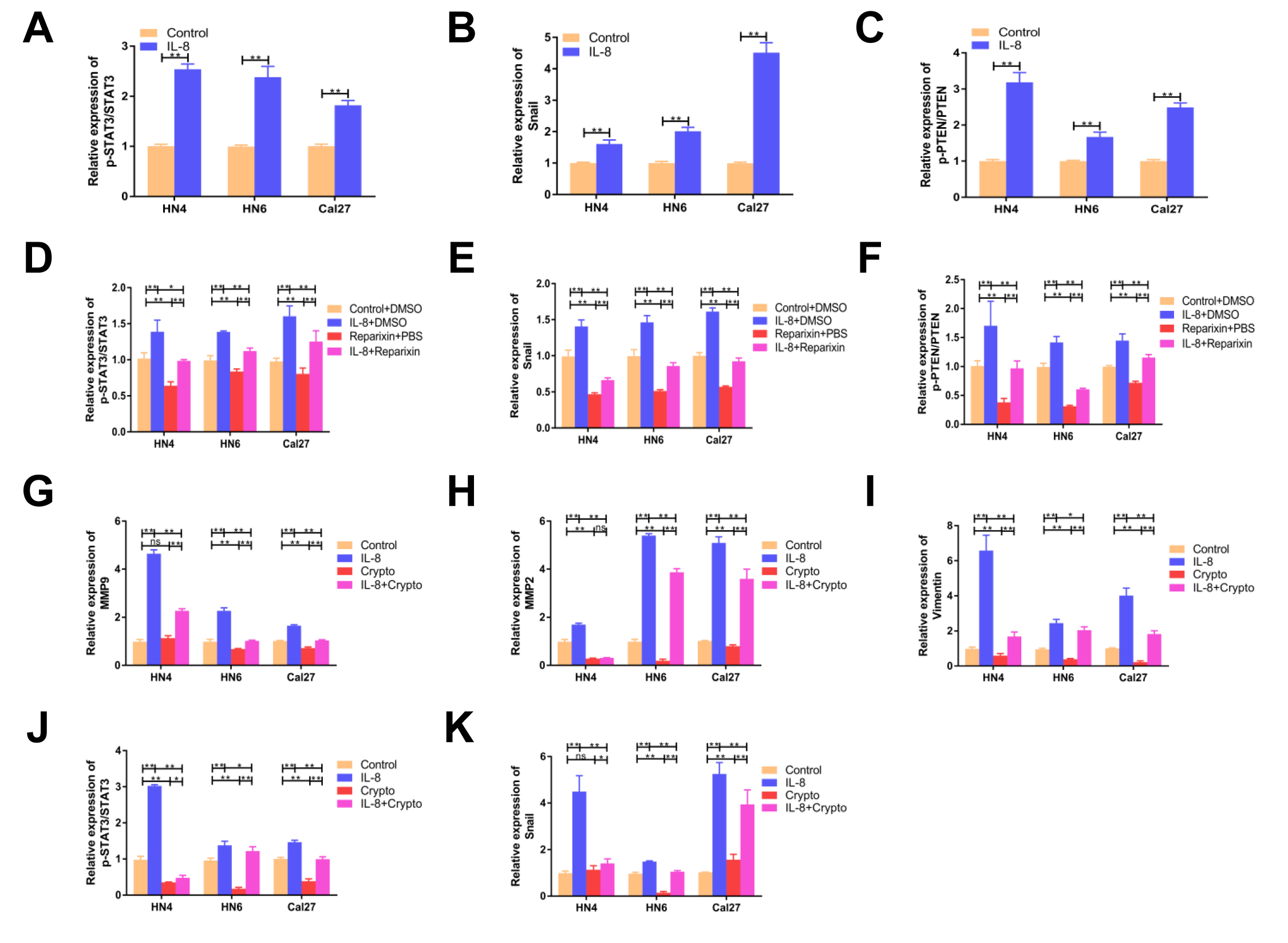

Supplement: Supplementary file 5 — Supplementary Figure 4 [file 41419_2020_2627_MOESM5_ESM.tif]

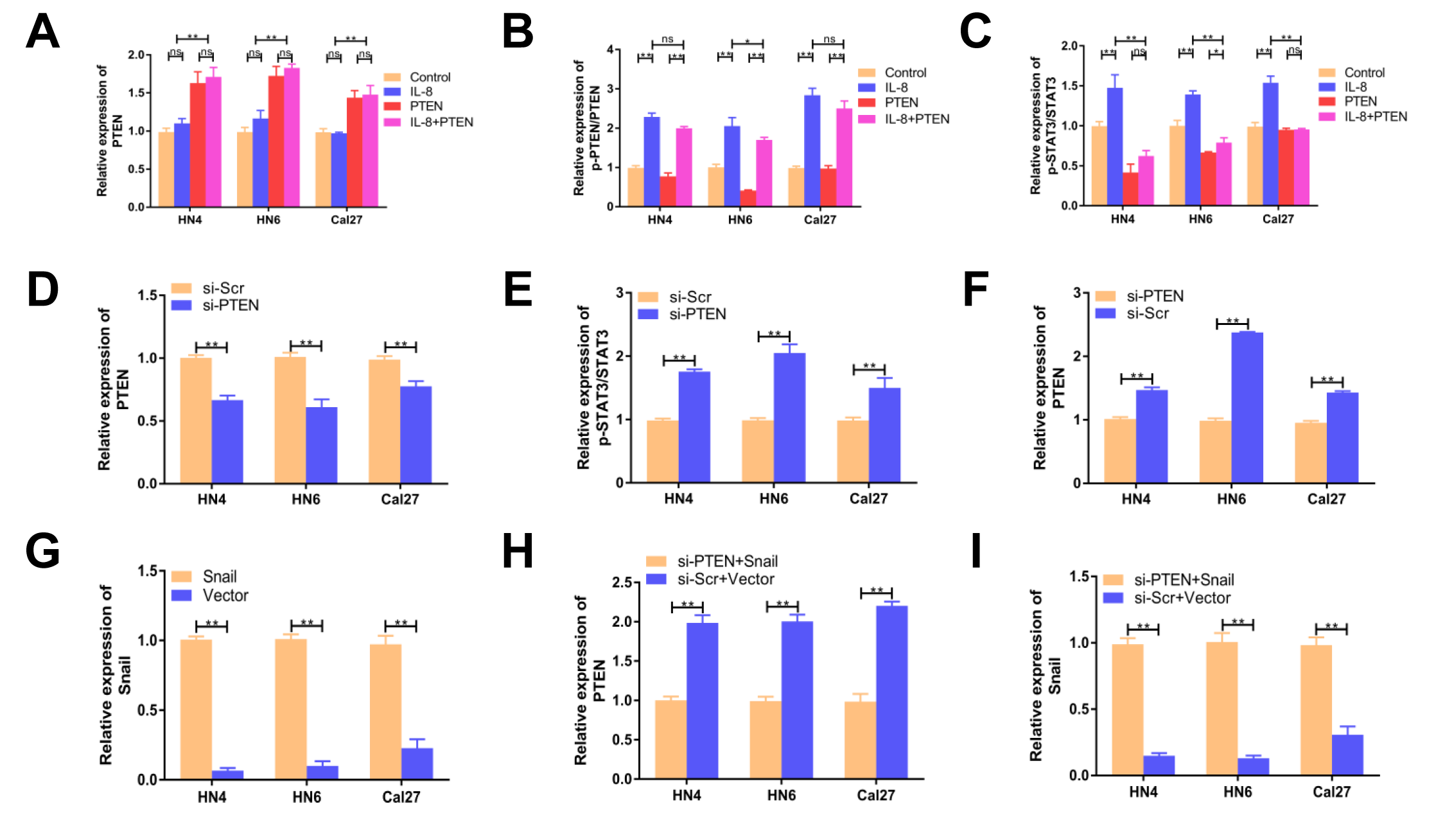

Supplement: Supplementary file 6 — Supplementary Figure 5 [file 41419_2020_2627_MOESM6_ESM.tif]

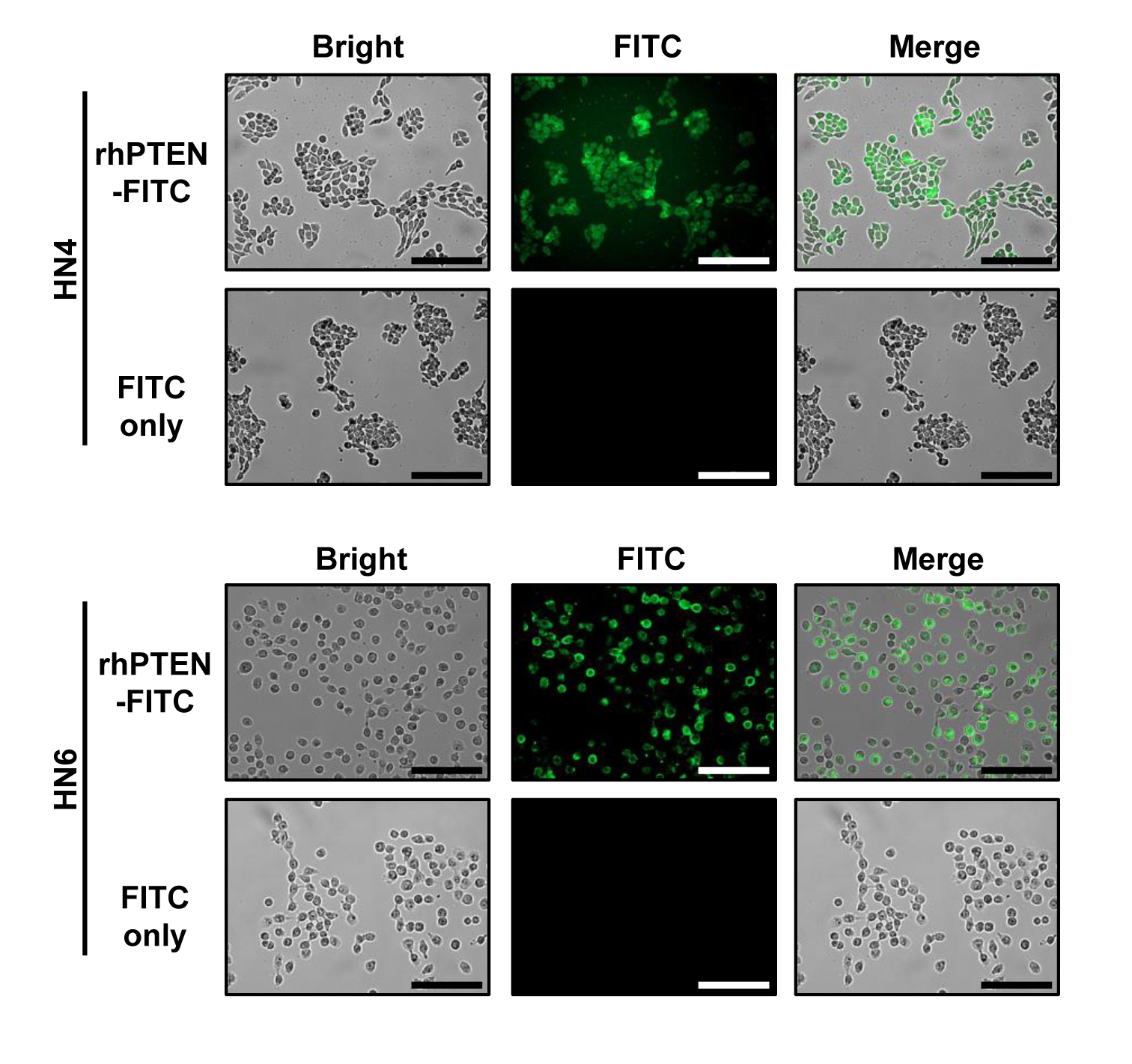

Supplement: Supplementary file 7 — Supplementary Figure 6 [file 41419_2020_2627_MOESM7_ESM.tif]

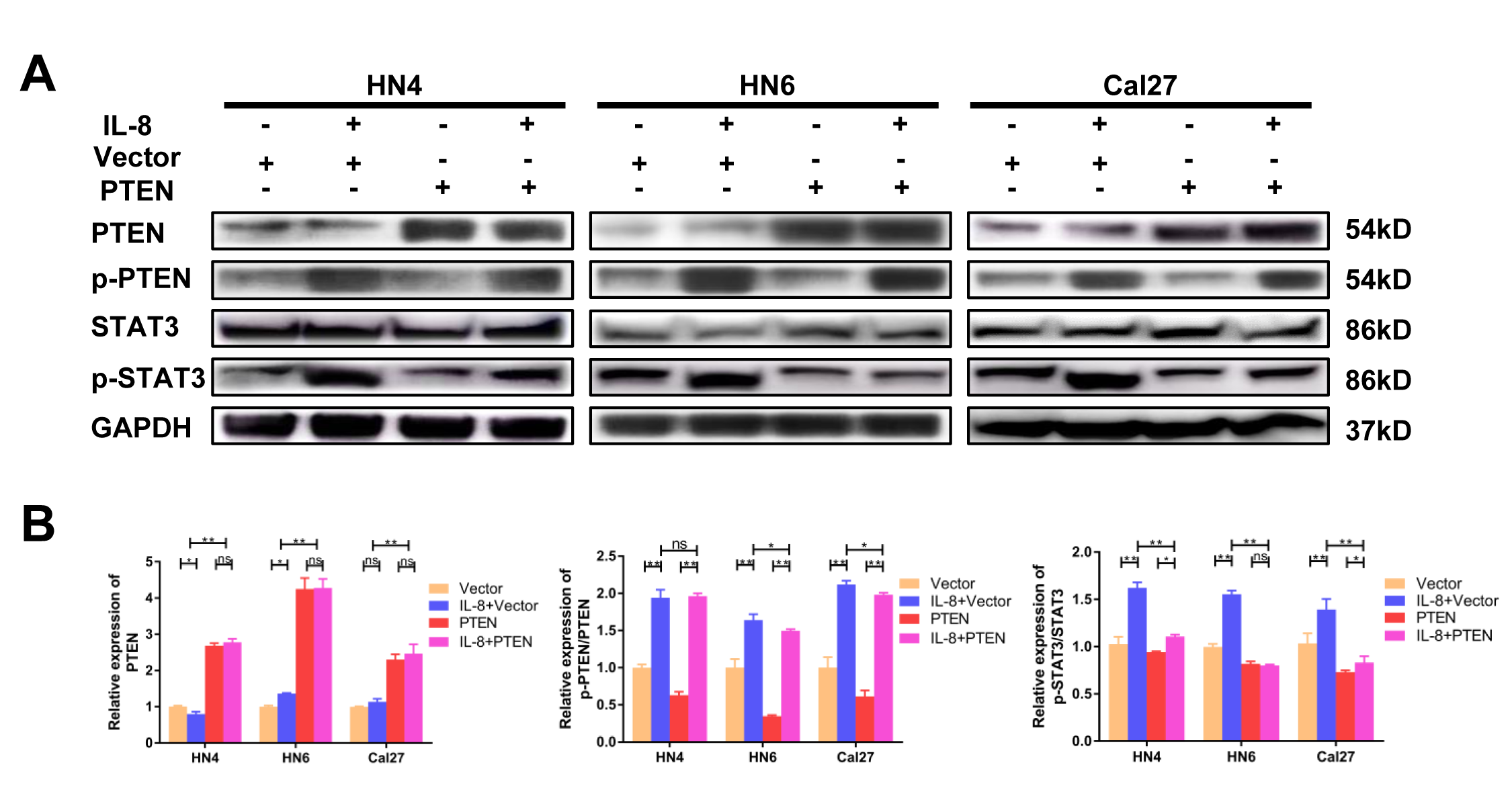

Supplement: Supplementary file 8 — Supplementary Figure 7 [file 41419_2020_2627_MOESM8_ESM.tif]
